# Updated cost-effectiveness and risk-benefit analysis of two infant rotavirus vaccination strategies in a high-income, low-endemic setting

**DOI:** 10.1186/s12916-018-1134-3

**Published:** 2018-09-10

**Authors:** P. Bruijning-Verhagen, J. A. P. van Dongen, J. D. M. Verberk, R. Pijnacker, R. D. van Gaalen, D. Klinkenberg, H. E. de Melker, M.-J. J. Mangen

**Affiliations:** 1 Department of Epidemiology and Surveillance, Centre for Infectious Diseases Control, National Institute of Public Health and the Environment, Bilthoven, The Netherlands; 2 Julius Centre for Health Sciences and Primary Care, University Medical Centre Utrecht, Utrecht, The Netherlands

**Keywords:** Rotavirus vaccination, Cost-effectiveness, Economic evaluation, Epidemiology, Risk-benefit, Intussusception, Risk factors, Prematurity

## Abstract

**Background and objective:**

Since 2013, a biennial rotavirus pattern has emerged in the Netherlands with alternating high and low endemic years and a nearly 50% reduction in rotavirus hospitalization rates overall, while infant rotavirus vaccination has remained below 1% throughout. As the rotavirus vaccination cost-effectiveness and risk-benefit ratio in high-income settings is highly influenced by the total rotavirus disease burden, we re-evaluated two infant vaccination strategies, taking into account this recent change in rotavirus epidemiology.

**Methods:**

We used updated rotavirus disease burden estimates derived from (active) surveillance to evaluate (1) a targeted strategy with selective vaccination of infants with medical risk conditions (prematurity, low birth weight, or congenital conditions) and (2) universal vaccination including all infants. In addition, we added herd protection as well as vaccine-induced intussusception risk to our previous cost-effectiveness model. An age- and risk-group structured, discrete-time event, stochastic multi-cohort model of the Dutch pediatric population was used to estimate the costs and effects of each vaccination strategy.

**Results:**

The targeted vaccination was cost-saving under all scenarios tested from both the healthcare payer and societal perspective at rotavirus vaccine market prices (€135/child). The cost-effectiveness ratio for universal vaccination was €51,277 at the assumed vaccine price of €75/child, using a societal perspective and 3% discount rates. Universal vaccination became cost-neutral at €32/child. At an assumed vaccine-induced intussusception rate of 1/50,000, an estimated 1707 hospitalizations and 21 fatal rotavirus cases were averted by targeted vaccination per vaccine-induced intussusception case. Applying universal vaccination, an additional 571 hospitalizations and <  1 additional rotavirus death were averted in healthy children per vaccine-induced intussusception case.

**Conclusion:**

While universal infant rotavirus vaccination results in the highest reductions in the population burden of rotavirus, targeted vaccination should be considered as a cost-saving alternative with a favorable risk-benefit ratio for high-income settings where universal implementation is unfeasible because of budget restrictions, low rotavirus endemicity, and/or public acceptance.

**Electronic supplementary material:**

The online version of this article (10.1186/s12916-018-1134-3) contains supplementary material, which is available to authorized users.

## Introduction

**Fig. 1:**
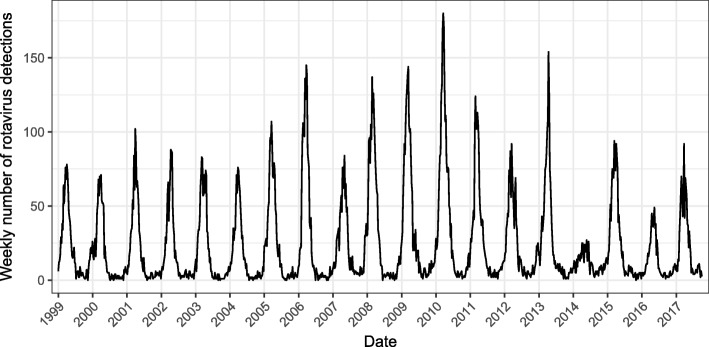
Weekly number of rotavirus detections in sentinel laboratory surveillance (for 2017 only up to week 40)

In recent years, the Netherlands has seen an unexpected change in rotavirus epidemiology, while infant rotavirus vaccination coverage (the vaccine has been licensed since 2006) has remained below 1%. Annual epidemics were observed until 2013; thereafter, an alternating pattern of high- and low epidemic years emerged (Fig. 1). During low endemic years, rotavirus detections in virological surveillance decreased by 58% (2014) and 52% (2016) compared to an average of the years before 2013, and a delayed start of rotavirus seasons was observed [1, 2]. Similarly, general practice (GP) consultation rates for acute gastroenteritis (AGE) during the winter months in children under 5 years old were reduced [3], and the prevalence of asymptomatic rotavirus observed in daycare attendees was significantly lower in 2014 (prevalence rate 0.6%) compared to 2011–2013 (prevalence rate 6.8–11.2%) [4]. Rotavirus detections and seasonal GP consultation rates during the alternating years 2015 and 2017 were comparable to pre-2014 numbers [3, 5]. Due to this changing epidemiology, the overall incidence of rotavirus disease in the Dutch pediatric population has reduced substantially. To our knowledge, a similar change in epidemic pattern has not been observed in any other European country without a national infant rotavirus vaccination program.

Although the driving factors for this change in epidemic pattern are currently unknown, it has been suggested that, apart from a declining birth rate and temperature fluctuations, rotavirus dynamics in the Netherlands may also be influenced by vaccination policies in neighboring countries [6]. Universal rotavirus vaccination programs have been implemented in all three neighboring countries of the Netherlands (Belgium in 2006, Germany in 2013, the UK in 2013), with coverage varying between 78% and 94% [7–9]. Implementation in these countries was followed by a sustained reduction in rotavirus detections of 44–75% [10]. This reduced circulation of rotavirus may have influenced the number of rotavirus introductions into the Netherlands.

We previously evaluated the cost-effectiveness of infant rotavirus vaccination in the Netherlands and considered three potential vaccination strategies: “no vaccination,” “universal vaccination,” and “targeted vaccination” [11]. The targeted vaccination strategy is a selective vaccination program, including only infants with medical risk conditions predisposing to severe or complicated rotavirus AGE, including prematurity, low birth weight, and severe congenital pathology [10]. No decision has been made yet on the preferred strategy for the Netherlands. Assuming the observed biennial pattern in rotavirus epidemics represents a new epidemiological equilibrium, a re-assessment of the national rotavirus disease burden and the cost-effectiveness of each of these infant rotavirus vaccination strategies is therefore required to inform policy makers. In addition, it is now widely recognized that rotavirus vaccination induces a small but increased risk of intussusception (IS). Because of this serious side effect, an evaluation of risk-benefit ratios has become an integral part of the decision-making process on rotavirus vaccination policy.

Our aim was to update our previous model-based health economic evaluation of rotavirus vaccination in the Netherlands by both taking into account the present, lower endemic state, and expanding the analysis by including risk-benefit calculations.

## Methods

### Updated rotavirus disease burden

The original economic evaluation [11] used data from three observational studies conducted in the Netherlands: (1) the Sensor cohort study on community AGE [12] and (2) the Netherlands Institute of Primary Health Care (NIVEL) study on AGE in primary care [13] were conducted in 1999 and provided age-stratified data on AGE incidence and the proportion rotavirus attributed; (3) the RoHo study quantified rotavirus community-acquired hospitalizations and nosocomial infections in children 0–15 years old for the years 2006–2010 with separate estimates for healthy children and those with medical risk conditions [11, 14]. Incidence estimates by disease category (rotavirus in the community, GP visits, community-acquired hospitalizations, and nosocomial infections) and by risk status (healthy vs medical risk group) were derived from these studies and used as input for the original cost-effectiveness model. To represent the average rotavirus disease burden over the period 2013–2016, covering two high and two low epidemic seasons, we updated these input parameters using the data sources and methodology as discussed in the following sections.

#### Rotavirus community incidence

Virological laboratory surveillance in the Netherlands collects weekly numbers of rotavirus detections from 17 to 21 sentinel laboratories serving primary care, hospitals, and long-term care facilities [5]. Time series analyses have confirmed that these surveillance data correlate well with rotavirus disease activity in the population [15, 16]. Rotavirus surveillance data were used to scale the community incidence of rotavirus AGE as originally measured in 1999 (Sensor study [12]) to the average for the years 2013–2016. We calculated the scaling factor as follows: 1 minus ([mean]annual rotavirus detections between 2013 and 2016/annual rotavirus detections in 1999). We kept the original rotavirus age distribution and the age-specific proportion of cases visiting primary care, as no updated estimates were available. The scaled incidence estimates were applied to the 2016 population size to obtain the annual expected total number of community cases and GP visits; see Table 1 [17].

**Table 1:** Parameters for model input

Parameter	Total population	Non-target group	Target group	Distribution	Data source	Method
Birth cohort *n* (%)	171,387	157,847 (92.1%)	13,540 (7.9%)	Fixed	Statistics Netherlands [46, 47]	Birth cohort size 2016. Prevalence of high-risk conditions, same as in Bruijning et al. [11]
Rotavirus incidence	*Most likely value (minimum–maximum)*					
Population: < 1 year	15,188 (10,161-21,597)	Calculated		Pert	Community-based cohort study [12]; virological rotavirus surveillance data [5]	Incidence based on simulations from original study data (for details see [17]), scaled to the years 2013–2017. Distribution among non-target and target groups based on relative size of each category in birth cohort
Population: 1–4 years	35,756 (21,805-54,972)					
Population: 5–14 years^a^	7897 (1426-26,004)					
GP visits < 1 year	21.5% (12.8–29.1%)			Pert	GP-based cohort study [13]	Percentage of rotavirus cases based on simulations from original study data (for details see [17])
GP visits 1–4 years	18.5% (16.3–20.8%)					
GP visits 5–14 years	6.4% (4.8–7.3%)					
Community-acquired (CA) hospitalization	2024 (1789 – 2256)	82.8% (82.7– 82.9%) of total	Calculated *(total minus non-target)*	Pert	RoHo study [14]; indirect estimated annual hospitalizations [2]; RIVAR AGE surveillance [18]	Incidence based on original study data (for details see [11]) and scaled to the years 2013–2016. Distribution over non-target and target groups and over CA and nosocomial cases based on active AGE surveillance in 2014–2016
Nosocomial infections per CA case	Calculated	0.21 (0.206–0.213)	0.89 (0.88–0.90)	Pert	RIVAR AGE surveillance [18]	
Mortality rate/1000 hospitalizations	Calculated	0.00 (0.00; 0.04)	0 81 (0.36; 1.46)	Pert	RoHo study [14]; External dataset Sophia Children’s hospital	For details see [11]
Age distribution of hospitalizations and fatal cases	See Additional file 1: Table S2 in Bruijning et al. [11]				RoHo study [14];	Same as [11]
Intussusception (IS) incidence						
Vaccine-induced IS risk	1/50,000 vaccinated children			Fixed	[32–35]	
Complicated (with intestinal resection)	4.8% of induced IS cases			Fixed	[36]	Calculated from [36]: 56 with resection out of 1176 IS cases in infants < 12 months
Utilities rotavirus AGE	QALY loss					
Mild (no medical care)	0.0011			Fixed	GP study in Canada [48]	50% of estimate for moderate, similar to [11, 17, 31, 49]
Moderate (GP visit only)	0.0022			Fixed	GP study in Canada [48]	
Severe (hospitalization)	0.0034			Fixed	Emergency-department study in UK [20]	
Nosocomial	Calculated	Calculated	Calculated		RoHo study [14]	Based on severity distribution, same as in [10]
Rotavirus fatal cases	Calculated	81.5 minus patient age at rotavirus infection	Simulated, assuming LE of 1; 20; 41.3 minus patient’s age with probability of 1/3 each	Uniform	Statistics Netherlands [46]; Expert panel [11]	For non-target group, based on average LE in the Netherlands. For target group, same as Bruijning et al. [11]
Utilities intussception	QALY loss					
Uncomplicated IS	0.0037			Fixed	Based on Reyes et al. [37]	
Complicated IS	0.0111			Fixed	Assumption	Assuming three times more severe than uncomplicated IS; see Additional file 1
Healthcare costs [?A3B2 show $9#?]rotavirus AGE						
No medical care	€0			Fixed		
Standard GP visit (€/unit)	€33				Dutch reference prices [22]	If GP attendance; home visit: Pert (0; 0.1; 0.1), standard GP visit: Pert (0.9; 0.9; 1.0), and GP telephone consultation: Pert (0; 0.97; 0.97); same as in [11, 17] Average cost/episode including antibiotics, oral rehydration solutions, and other prescribed drugs/GP consultation (home or standard GP visit) Additional GP consultations for hospitalized cases same as in [11], based on [50]
GP home visit (€/unit)	€50					
GP telephone consultation (€/unit)	€17				Based on cohort studies [12, 13, 51]	
Drug costs incl. Prescription fee (€/unit)	€43					
Laboratory costs (€/unit)	€78				Expert elicitation	10% with laboratory test [52]
Ambulance (€/unit)	€618.6			Fixed	Dutch reference price [22]; hospital-based observational study [50]	1% of hospitalized cases transported by ambulance [50]
Rotavirus hospitalization (€/hospitalization)	Calculated	€2417 (2248–2584)	€2828 (2782–4000)	Pert	RoHo study [11]	Weighted estimates from original study data (see additional file in [11])
Nosocomial rotavirus (€/hospitalization)	Calculated	€2413 (1378–3048)	€2361 (1334–3388)			
Uncomplicated IS (€/hospitalization)	€1423			Fixed	Hospital administrative data (see Additional file 1); Valk et al. [53]	Average LOS for Dutch IS cases = 2.11 days + costs of diagnostics (i.e., abdominal X-ray, ultrasonography)
Complicated IS (€/hospitalization)	€6759			Fixed	Assumption	3× LOS for uncomplicated IS, whereof 1 day in ICU, and additional procedures (i.e., ileocecal resection, abdominal X-ray, ultrasonography). See Additional file 1
Patient and family costs for rotavirus AGE^b^						
Without medical care	Additional diapers			Uniform	Assumption	For details see [11, 17]
Requiring GP visit	Additional diapers and travel costs			Pert	Assumptions and guidelines for health economic evaluation [22]	
Hospitalization	Travel costs					
Nosocomial rotavirus	Not applicable					
Productivity losses caregiver						
Cost per hour *paid* work loss	€32				Statistics Netherlands [54] and guidelines for health economic evaluation [22]	
*Hours of paid work loss per episode:*						
Without medical care	1 day (~ 8 h) in 5% of episodes			Beta	RotaFam (see Additional file 1)	For children > 10 years of age work loss estimates were reduced by 50%
Requiring GP visit	0.5–2 days in 25% of episodes			Beta; uniform	RotaFam (see Additional file 1)	
Hospitalization	26.40				Based on [50]	For details see [17]
Nosocomial AGE	24.58				Based on [50]	For details see [11]
Uncomplicated IS	4.93			Fixed	Estimated based on LMR data (see Additional file 1) and Statistics Netherlands [54]	Based on LOS and applying average caregiver employment of 16.4 h/week (similar to Mangen et al. [38])^d^
Complicated IS	14.79			Fixed		
Vaccine coverage	Universal vaccination	Targeted vaccination				
Vaccine coverage	86.2%	86.2%		Fixed	Discrete choice experiment [28]	
Vaccine efficacy	Table 2 in Bruijning et al. [11]			Pert	Vaccine trials [55–57]	
Herd protection	See Table 2	Not applicable		Fixed	Published estimates, see Table 2	Only for universal vaccination scenarios
Vaccination costs						
Vaccine costs/infants^c^	€75	€135.32		Fixed	Free market price for targeted vaccination [29]; for universal vaccination based on assumption as in [11, 31]	
Application and administration costs	€12.36	€12.36		Fixed	[30]	
Start-up cost first year	€233,760			Fixed	[17]	

*LOS* length of hospital stay, *LE* life expectancy, *RIVAR* Risk-Group Infant Vaccination Against Rotavirus, *LMR* Netherlands National Medical Registry

^a^Of which 80% is aged 5–9 years and 20% is aged 10–14 years

^b^Note, we did not consider any patient and family costs for IS cases

^c^Reported vaccine costs exclude costs for spillage; 2% spillage costs was added in the model

^d^Based on population statistics for the year 2014 [54], the most recent year available, we calculated similarly as in Mangen et al. [38] the average working hours/week for a primary caregiver. For this we assumed that, except for single-father households, the female is the primary caregiver taking care of a sick child. In 2014 73.4% of primary caregivers had paid employment, for an average of 22.3 h/week. For an average primary caregiver in the Netherlands this corresponds to 16.4 h/week

#### Rotavirus hospitalizations

A similar approach was used to scale the annual number of community-acquired and nosocomial rotavirus hospitalizations from the RoHo study (2006–2010) to the average for the years 2013–2016. To calculate the scaling factor, we used virological surveillance data on annual rotavirus detections and annual AGE hospitalization data derived from inpatient primary and secondary discharge diagnoses collected by the Dutch National Medical Registry (LMR, national coverage around 90%). The anonymized discharge diagnoses were coded according to the 9th International Classification of Diseases (ICD-9) from 2001 up to 2012 and according to ICD-10 from 2013 onwards. Using an indirect method [15], the proportion of AGE-coded hospitalizations attributable to rotavirus (including community-acquired and nosocomial infections) was calculated for each year in children younger than 5 years. A scaling factor was then calculated from the indirectly estimated annual rotavirus hospitalizations comparing the mean of 2006–2010 (RoHo-study years) to the mean of 2013–2016. This scaling factor was applied to the mean annual number of rotavirus hospitalizations used in the original model (Table 1).

The proportion of rotavirus hospitalizations attributable to nosocomial or community-acquired infections and also the ratio of healthy vs risk-group children were originally derived from the RoHo study. Proportions were updated based on results from active AGE surveillance conducted in 12 Dutch hospitals between November 2014 and November 2016 [18, 19]. Collected data include age, sex, rotavirus presence in stool, type of infection (community-acquired or nosocomial), and the presence of medical risk conditions. As the active surveillance only included children < 2 years of age, proportions for older children were kept consistent to what was found in the RoHo study.

### Other parameter updates

Each model input parameter and assumption was checked for potential updates by screening the literature and checking available data from ongoing surveillance. As we outline in this section, this yielded new and improved data on the impact of rotavirus disease and vaccination, and we updated our parameters accordingly.

A recent UK study estimated the quality-adjusted life year (QALY) loss due to severe rotavirus AGE [20]. These estimates were applied as QALY loss for community-acquired rotavirus hospitalizations (Table 1).

For rotavirus episodes without medical care and those requiring GP visits, we updated our previous estimates on parental productivity losses due to work absence based on results from a prospective household study on AGE among 289 Dutch families with young children conducted between January and May 2016 [21]. (See Table 1 and Additional file 1 for details.)

All costs — healthcare costs, patient and family costs, and productivity losses — were updated to 2016 cost prices using Dutch consumer price indexes and recent reference prices (Table 1) [22].

Herd protection as a result of infant rotavirus vaccination, where rotavirus AGE in unvaccinated children is reduced, has been widely observed post-implementation in high-income, high-coverage settings [23–27]. We therefore incorporated herd-protection effects in our base case for universal infant rotavirus vaccination. We stratified herd-protection levels by age and by vaccinated vs unvaccinated cohorts (Additional file 1: Table S1). Unvaccinated age cohorts were assumed to be ineligible for vaccination based on age at the time of implementation, but may still benefit from herd effects. The available studies on herd-protection levels used historical pre-vaccination cohorts as a comparator in settings where annual rotavirus epidemics occurred [23–27]. To account for the presence of a biennial epidemic pattern in the current pre-vaccination setting in the Netherlands, we lowered study estimates by 50% for our analysis. This assumes that relevant reductions due to herd effects only occur every other year. We assumed no effect on adult rotavirus infections from any of the infant vaccination strategies [11] and no herd effects for targeted vaccination, as this would result in a maximum vaccine coverage of 8% in the infant population [11].

Further parameter updates included changing the vaccination coverage for both targeted and universal vaccination from 88% (vaccine coverage Belgium [11]) to 86% based on a recent assessment of willingness to vaccinate among Dutch parents [28], changing the vaccine costs for a targeted vaccination strategy to the current market price of €135.32 per child [29] and changing the application costs to €12.36 per dose [30]. Vaccine costs for a universal vaccination within the national immunization program were kept at €75 per child, which assumes that tender processes will lower vaccine prices by almost 50% [11, 31].

### Intussusception

Our previous model [11] was extended to include the risk of developing IS following rotavirus vaccination. Based on the available literature, we assumed a vaccine-induced IS rate of 1:50,000 [32–35], whereof 4.8% would result in complications ([36], Table 1). The associated QALY loss for uncomplicated IS was 0.0037 [37], and costs were based on the average length of stay (LOS; 2.11 days) for IS in the Netherlands (Table 1, see Additional file 1 for details). Threefold higher estimates, representing the 95% percentile of the LOS distribution, were used for complicated IS cases (see Additional file 1 for details). Parental work loss was based on LOS, and we assumed that an average caregiver works 16.4 h/week, based on the mean weekly workhours among the primary caregivers according to Statistics Netherlands in 2014 [38].

### Model

**Fig. 2:**
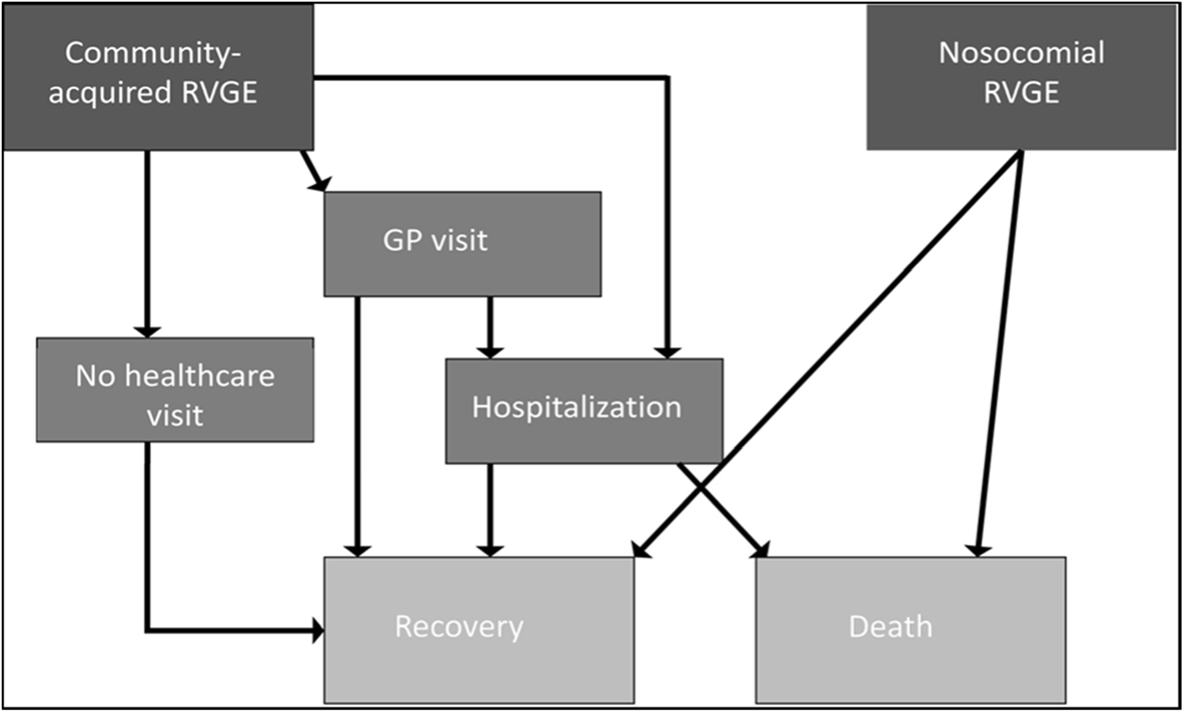
Rotavirus outcome tree and different healthcare paths considered in model. With permission from Bruijning-Verhagen et al. [11]

The model has been described previously [11]; see Fig. 2. In brief, we used an age- and risk-group structured, discrete-time event, stochastic multi-cohort model of the Dutch pediatric population. The model used separate estimates for the number, and the costs of, community-acquired and nosocomial rotavirus cases, stratified by risk stratus into healthy vs medical risk conditions, the latter qualifying for targeted vaccination (Table 1). The effect of either targeted or universal infant vaccination was modeled as a reduction in rotavirus AGE and associated health outcomes in vaccinated and non-vaccinated age cohorts between 0 and 15 years old, stratified by risk status. Time steps of 1 month were used for ages 0 to 11 months and time steps of 1 year for ages 1 to 15. A time horizon of 20 years was used with year 1 being the start of either vaccination program.

### Cost-effectiveness and risk-benefit analyses

The model was built in Microsoft Excel with the add-in software @Risk, version 7.5 (Palisade). For all three vaccination strategies (“no vaccination,” “targeted vaccination,” and “universal vaccination”), the model estimates the number of rotavirus cases in the population, GP visits, hospitalizations, rotavirus-related deaths, QALYs, and life years. The model further estimates the number of vaccine-induced IS cases and associated QALYs. Net costs (i.e., net social costs and net healthcare costs), life years gained (LYG), and QALYs gained were calculated by summing all costs, life years, and QALYs over the 20-year time horizon. For each simulation, 5000 runs were conducted using Monte Carlo sampling, accounting for the uncertainty of the model parameters (Table 1).

The incremental cost-effectiveness ratios (ICERs) were calculated by dividing the net cost differences between each vaccination strategy, compared to no vaccination, by either QALYs gained or LYG. Our primary perspective was societal (i.e., including non-healthcare costs such as caregiver work loss), and the healthcare payer perspective was included in the sensitivity analysis. Costs are expressed in 2016 euros. A discount rate of 3% was used for both costs and effects [39].

Risk-benefit ratios were calculated by dividing the number of severe outcomes averted by vaccination, which included rotavirus hospitalizations or rotavirus fatal cases, by (1) the estimated number of vaccine-induced IS cases and (2) the estimated number of vaccine-induced complicated IS cases. The calculated ratios were used to obtain the benefit per vaccine-induced IS case and per vaccine-induced complicated IS case, respectively. Risk-benefit ratios were calculated both for the total population and for each risk group, since the risk of severe outcomes due to rotavirus differs between children with and without medical risk conditions.

### Sensitivity and scenario analyses

Univariate sensitivity analysis was conducted to identify critical parameters driving our results. In short, parameter variations included 25% lower and 25% higher rotavirus hospitalization rates and hospitalizations costs; vaccine-induced IS rates of 1:20,000 and 1:100,000 (base case 1:50,000) [32–35], and IS complication rates of 0% and 9.6% (base case 4.8%). We also included slightly higher QALY losses based on the sensitivity analysis of Marlow et al. (for hospitalizations 0.0039 vs 0.0030 and for GP visits 0.0030 vs 0.0022) [20]) As caregiver work-loss estimates for rotavirus AGE are influenced by local employment conditions and parental leave plans, they can vary substantially by country. Our sensitivity analysis therefore also included 100% higher caregiver productivity losses. Subsequently, we tested the impact of old vs new parameter estimates including caregiver work loss for mild and moderate rotavirus cases [4] and QALY losses for hospitalized cases [11]. We applied various discount rates: 2% and 4% for both costs and effects (3% in the baseline), as well as the Dutch discount rates (1.5% for effects and 4% for costs [10]). Extensive sensitivity analyses were conducted on vaccine costs to determine the thresholds at which the vaccination strategies would become cost-saving under base-case assumptions.

Additionally, strategy-specific scenarios included the following: a lower vaccination coverage of 75% for “targeted vaccination” (baseline 86%); decreased or increased herd protection, or no herd protection at all in case of universal vaccination. Because a shift to a biennial rotavirus epidemic pattern could theoretically increase the average age of first infection as a result of the reduced force of infection, we assessed the impact of an “older” age when first infected. To this end, we simulated scenarios where 50% or 75% of the 0–1 years old patients with rotavirus from baseline were 1–2 years old instead, and consequently had lower probabilities of seeking medical care, both GP and hospitalization. Finally, an “alternative universal vaccination” scenario was also analyzed where we assumed that “universal vaccination” would be recommended, but not covered by the publicly funded national immunization program. Instead, vaccines would be individually purchased for each infant with or without partial reimbursement from health insurance. For this scenario, we assumed a coverage of 60%, no herd protection due to the lower coverage, and the actual market price (i.e., €135.32/child). For more details see also Additional file 2: Tables S2 and S3.

## Results

The updated rotavirus disease burden estimated a reduction in the number of rotavirus AGE episodes in the Netherlands by 13% compared to 1999, and in the number of hospitalizations by 45% compared to 2006–2010. The 2014–2016 active surveillance data identified a somewhat higher proportion of children with medical risk conditions (26% vs 16%) among those < 2 years of age hospitalized for rotavirus and a higher proportion of nosocomial infections (28% vs 11%) compared to the RoHo study [11].

Without vaccination and over a 20-year time horizon, an estimated 1.25 million rotavirus AGE episodes (62,500 annually), 54,000 hospitalizations (2700 annually), and 110 fatal rotavirus cases (5.5 annually) in children 0–15 years old would occur in the Netherlands, resulting in 2597 QALYs lost (130 annually) or 1309 life years lost (65.45 annually), and in societal costs of €180 million (Є9 million annually; see Table 2).

**Table 2:** Rotavirus disease and cost burden in children < 15 years old (mean (95% credibility interval) and incremental results from targeted or universal infant rotavirus vaccination based on a 20 years’ time horizon

Disease and cost burden	AGE episodes (× 1000)	Hospitalizations^a^ (× 1000)	Fatal cases	Vaccine- induced IS	QALYs lost^b^	Life years lost^b^	Net societal costs^b^ (mio €)	
No vaccination	1251 (903–1627)	54 (48–60)	110 (59–175)	NA	2597 (1681–3727)	1309 (471–2372)	180 (153–218)	
Targeted vaccination	1208 (871–1573)	46 (41–51)	12 (5–23)	4.61	1458 (1057–1890)	195 (18–463)	163 (139–199)	
Universal vaccination	586 (407–789)	14 (12–16)	7 (4–11)	58.40	689 (477–923)	105 (5–245)	278 (268–294)	
Incremental results from vaccination	Averted AGE episodes (× 1000)	Averted hospitalizations (× 1000)	Averted fatal cases	Additional IS	Incremental QALYs gained	Incremental life years gained	∆ net societal costs (in mio €)^c^	ICER €/QALY gained
Targeted vaccination vs *no* vaccination								
Absolute change	433 (32–55)	8 (7–9)	99 (54–153)	4.61	1139 (426–2022)	1114 (399–2004)	-−17^**c**^ (−21 to –13.6)^**c**^	Cost-saving (cost-saving − cost-saving)
Percent reduction	3.4% (2.9–4.0%)	14.7% (13.9–15.3%)	89.8% (86.7–92.2%)	NA	42.7% (23.0–57.7%)	85.6% (72.3–97.2%)	9.4% (8.0–11.0%)	NA
Universal vaccination vs *no* vaccination								
Absolute change	664 (482–864)	40 (35–45)	103 (56–165)	58.40	1907 (1114–2915)	1204 (428–2191)	98 (74–116.4)	51,277 (29,259–94,686)
Percent reduction	53.2% (48.1–58.4%)	74.4% (71.9-76.5%)	93.9% (92.7–94.8%)	NA	72.9% (63.0–81.1%)	92.1% (82.5–99.3%)	NA	NA
Universal vaccination vs *targeted* vaccination^d^								
Absolute change	622 (451–810)	32 (28–36)	4 (1–12)	53.79	769 (561–1003)	90 (9–239)	115 (94–131)	149,282 (101,101–220,113)
Percent reduction	51.5% (46.5–56.7%)	70% (67.4–72.3%)	39.5% (26.9–53.4%)	NA	52.8% (47.6–58.8%)	48.2% (25.4–88.6%)	NA	NA

^a^Including nosocomial infections

^b^Using a 3% discount rate for effects (QALYs/life years) and costs

^c^Negative costs are savings

^d^Comparing universal vaccination to targeted vaccination in order to obtain the incremental results of extending targeted vaccination to universal vaccination

**Fig. 3:**
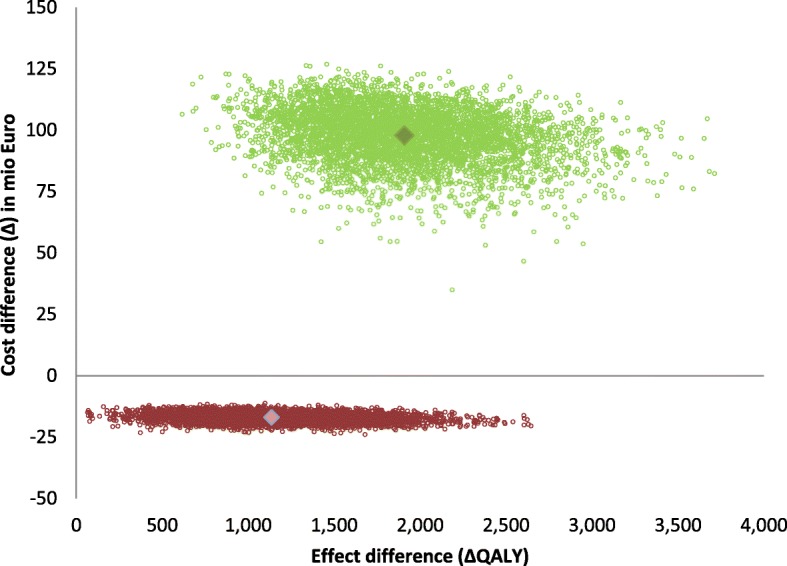
Cost-effectiveness plane for targeted vaccination (*depicted in red*) and universal vaccination (*depicted in green*) using a societal perspective and a 3% discount rate

**Fig. 4:**
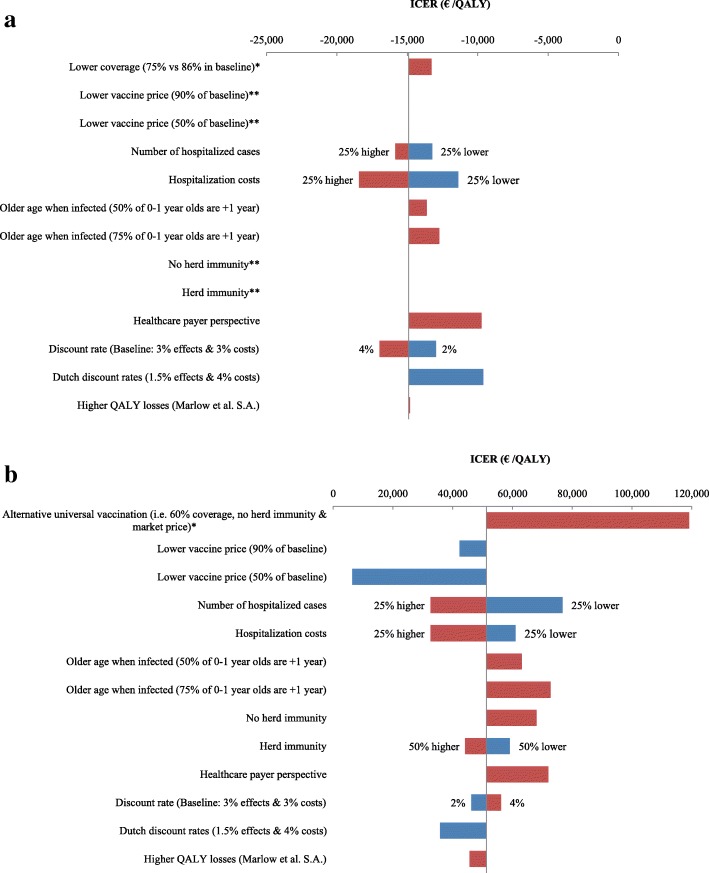
Tornado diagram showing one-way and multi-way sensitivity and scenario analyses results for **a** a targeted vaccination strategy and **b** a universal vaccination strategy Note1: The x-axis shows the effect of changes in selected variables on the mean incremental cost-effectiveness ratio (ICER) for the base-case taking a societal perspective. The y-axis shows the model parameter that was varied. The bars indicate the mean change in the ICER caused by changes in the value of the indicated variable holding all other parameters similar, whereby a blue bar indicates a lower value of the selected variable(s) as in the baseline and a red bar a higher value of the selected variable(s). Sensitivity analyses with less than 5% changes are not shown. Detailed results are presented in Table S2 in Additional file 2 for targeted vaccination and in Table S3 in Additional file 2 for universal vaccination. Note2: All scenarios for targeted vaccination were cost-saving and health gaining. This results in negative ICERs. *Some of the sensitivity analyses were only applicable to universal vaccination (i.e. alternative universal vaccination strategy), and others were only to target vaccination (i.e. lower coverage in the target population). **No S.A. on vaccine price was performed for targeted vaccination as this was already cost-saving at the current market price; No S.A. on herd immunity, as a population vaccine coverage of 7% will not induce herd protection

We first compared targeted vaccination to no vaccination over a 20-year time horizon. With annual vaccination costs of €0.64 million, targeted vaccination would avert on average 43,000 rotavirus AGE episodes and 99 fatal cases, and would induce 4.6 IS cases, of which 0.22 would be complicated cases. The targeted vaccination strategy would result in 1139 QALYs gained and €17 million savings (Table 2). Targeted vaccination was cost-saving in all simulations (Fig. 3) and remained cost-saving in all conducted sensitivity analyses (Fig. 4a and Additional file 2: Table S2).

**Fig. 5:**
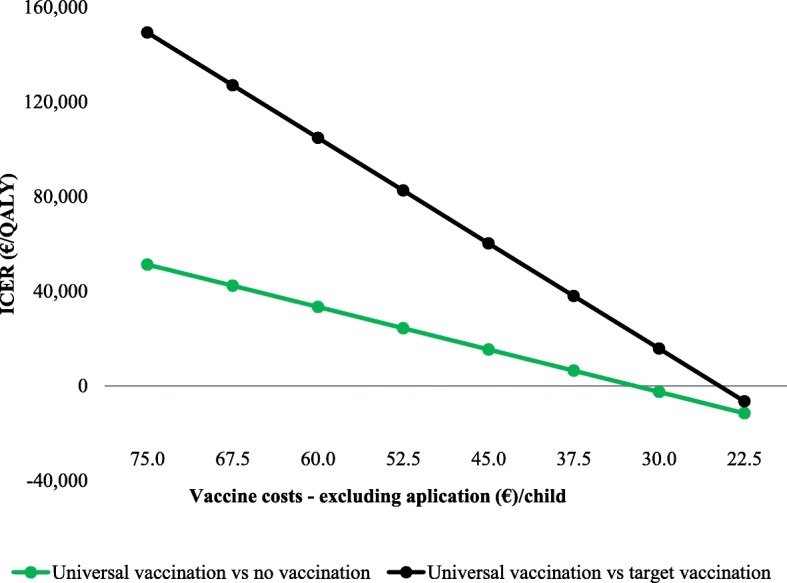
Mean ICER (cost per QALY gained) for universal vaccination vs no vaccination (*green line/dots*), and for universal vaccination vs targeted vaccination (*black line/dots*) using a societal perspective and assuming a discount rate of 3%, for different vaccine costs. Results are also presented in Table S3 in Additional file 2 (universal vaccination vs no vaccination) and in Table S4 in Additional file 2 (universal vaccination vs targeted vaccination)

We then compared the no vaccination strategy to universal vaccination, which would cost €15 million annually. Over a 20-year time horizon universal vaccination would avert 665,000 rotavirus AGE episodes and 103 fatal cases and would induce 58.4 IS cases, of which 2.8 would be complicated. Universal vaccination would result in 1907 QALYs gained and €98 million additional costs (Table 2) at an ICER of €51,280/QALY gained (Fig. 2 and Additional file 2: Table S3). When universal vaccination was compared to targeted vaccination, the ICER increased to €149,280/QALY gained. Sensitivity analyses revealed that vaccine costs, presence and level of herd protection, the perspective chosen (i.e., healthcare costs only vs societal costs), the number of annual rotavirus hospitalizations, the costs per hospitalization, older age at first infection, and productivity losses were most influential on cost-effectiveness results (Figs. 4, 5 and Additional file 2: Table S3 and Figure S1). Under base-case assumptions and using a societal perspective, universal vaccination would become cost-saving at vaccine costs of €32 per child when compared to a strategy with no vaccination, or at €24.5 per child when compared to a strategy with targeted vaccination. The alternative universal vaccination scenario, where the vaccine would not be covered by the publicly funded national immunization program but purchased individually at market prices, was not considered cost-effective at an ICER of €119,191/QALY (95% credibility interval (CI) €70,488/QALY–€244,692/QALY) (see Fig. 3 and Additional file 2: Table S3).

Vaccination averts fatal rotavirus cases and hospitalization (benefits), but at the costs (risk) of inducing IS cases. The risk-benefit ratio differed by health status of the vaccinated child (Table 3). Among infants with medical risk conditions, we estimated a benefit of 21 prevented fatal rotavirus cases and 1707 prevented rotavirus hospitalizations for every vaccine-induced IS case. In healthy children the estimated risk-benefit ratio resulted in 0.05 prevented fatal cases and 571 hospitalized cases for every vaccine-induced IS case.

**Table 3:** Risk-benefit ratios for rotavirus vaccination

	Induced IS: prevented fatal cases	Induced IS: prevented hospitalized cases	Induced complicated IS: prevented fatal cases	Induced complicated IS: prevented hospitalized cases
All children				
Targeted vaccination	1:21 (1:12–1:33)	1:1707 (1:1494–1:1920)	1:445 (1:244–1:691)	1:35,564 (1:31,126–1:39,995)
Universal vaccination	1:1.8 (1:1.0–1:2.8)	1:685 (1:603–1:767)	1:37 (1:20–1:59)	1:14,267 (1:12,566–1:15,974)
Targeted group				
Targeted vaccination	1:21 (1:12–1:33)	1:1707 (1:1494–1:1920)	1:445 (1:244–1:691)	1:35,564 (1:31,126–1:39,995)
Universal vaccination	1:22 (1:12–1:34)	1:2012 (1:1773–1:2252)	1:455 (1:250–1:706)	1:41,913 (1:36,942–1:46,921)
Healthy children				
Targeted vaccination	NA	NA	NA	NA
Universal vaccination	1:0.05 (1:0.00–1:0.16)	1:571 (1:503–1:639)	1:1.0 (1:0.03–1:3.24)	1:11,896 (1:10,475–1:13,319)

## Discussion

Our results show that, in a high-income and relatively low rotavirus endemic setting, targeted rotavirus vaccination of infants with medical risk conditions is a cost-saving strategy and has the most favorable risk-benefit ratio. This finding remains robust in all of our sensitivity analyses. This strategy would also nearly eliminate rotavirus-related mortality in high-income settings, where fatal rotavirus cases among otherwise healthy children are extremely rare. Yet, the impact of targeted vaccination on the rotavirus disease burden in the pediatric population is limited, with only a 3.4% reduction in AGE episodes and a 14.7% reduction in hospitalizations (Table 2).

Universal rotavirus vaccination has the potential to reduce the population rotavirus disease burden in children by > 50% and avert nearly 75% of hospitalizations (Table 2). However, in a low-endemic setting and at assumed vaccine costs of €75 per child, the ICER for universal rotavirus vaccination at €51,280/QALY for the societal perspective and at €72,021/QALY for the healthcare perspective is not considered a cost-effective intervention according to most internationally accepted willingness-to-pay thresholds [40–42]. Further reductions in vaccine prices are therefore needed to improve cost-effectiveness. Universal vaccination could become cost-saving when vaccine costs are reduced to €32 per child or less. Importantly, even in a low-endemic setting, the risk-benefit ratio for healthy children vaccinated under a universal vaccination strategy can still be considered favorable at 571 averted hospitalizations for every vaccine-induced case of IS.

Our analysis also showed that the alternative universal vaccination scenario, i.e., no publicly funded program but vaccines individually purchased, is the least favorable strategy due to higher vaccine costs per child (no price reductions generated through tender processes) and absence of herd protection because of moderate vaccine uptake. Yet, this or comparable strategies are currently in use in several high- or middle-income countries [43]. Health authorities may therefore wish to reconsider one of the alternative, more cost-effective vaccination strategies.

Healthcare budget restrictions and prioritization may be an important reason why a publicly funded universal vaccination program is unfeasible. In this situation, a publicly funded targeted vaccination program can form a suitable alternative, as it results in cost savings both from the societal and healthcare payer perspective, while protecting the most vulnerable infants. Concerns about vaccine safety of the currently licensed vaccines and public acceptance may be another reason for not implementing universal vaccination. For instance, in France several reports on severe and even fatal IS cases following rotavirus vaccination resulted in public concern and the decision by health authorities to withdraw the recommendation for routine infant rotavirus vaccination [44]. A recommendation for targeted vaccination could offer an acceptable solution because of the more favorable risk-benefit ratio.

Our study has several limitations. The model input was largely based on epidemiological data as well as healthcare and non-healthcare cost estimates from the Netherlands. Differences in rotavirus endemicity, population demographics, caregiver employment, and cost prices may limit the generalizability of our findings to other high-income settings. However, we have performed extensive sensitivity analyses to evaluate the robustness of our ICER estimates and the most influential parameters. As targeted vaccination remained cost-saving under all scenarios tested, we are confident that this strategy will be cost-saving to other high-income settings. The ICER for universal vaccination, however, may be more variable, and for some high-income countries it may be better represented by one of the alternative scenarios from our sensitivity analysis.

Our model did not include dynamic simulation of herd effects following introduction of universal vaccination. Given the unusual pre-vaccination biennial rotavirus pattern in the Netherlands, observations on herd-protection levels from other countries may not be representative. Therefore, we chose to lower the herd-protection estimates extracted from studies in Europe and North America by 50% for our analysis. We considered this the most likely scenario, but the accuracy of these adjusted estimates remains uncertain. Our sensitivity analysis showed that a 50% change in herd effects from baseline would result in a 15% change in ICER. Another limitation of our static, rather than a dynamic model, is that we could not explore how universal rotavirus vaccination affects the timing and pattern of rotavirus epidemic peaks. Sudden spikes in incidence put additional pressure on hospital capacity, and this may be especially relevant if these coincide with circulation of respiratory viruses in winter months. The periodicity and timing of rotavirus epidemics may therefore be important for bed capacity planning. Available rotavirus dynamic models so far suggest that high-coverage rotavirus vaccination in temperate climates results in a biannual pattern and a shift of the epidemic peak to April/May [45].

Finally, it is currently uncertain whether the biennial rotavirus pattern in the Netherlands will be sustained in future years. If conditions affecting rotavirus epidemiology change in the future, disease levels could return to those pre-2014. Naturally, this would change the ICERs for the different vaccination strategies analyzed and the threshold for cost-saving vaccine prices.

## Conclusion

While universal infant rotavirus vaccination results in the highest reductions in the population burden of rotavirus, targeted vaccination should be considered as a cost-saving alternative with the most favorable risk-benefit ratio for high-income settings where universal implementation is unfeasible for reasons of budget restrictions, low rotavirus endemicity, and/or public acceptance.

## Additional files

Additional file 1: Model input data. (DOCX 27 kb)

Additional file 2: Additional results. (DOCX 71 kb)

## Abbreviations

AGE: Acute gastroenteritis
CI: Credibility interval
GP: General practice
ICER: Incremental cost-effectiveness ratio
IS: Intussusception
QALY: Quality-adjusted life year
