# Supplementary material for: Updated cost-effectiveness and risk-benefit analysis of two infant rotavirus vaccination strategies in a high-income, low-endemic setting
Source: BMC Med. 2018 Sep 10;16:168. doi: 10.1186/s12916-018-1134-3 (PMC6130096; doi:10.1186/s12916-018-1134-3)
Supplement: Supplementary file 1 — Model input data. (DOCX 27 kb) [file 12916_2018_1134_MOESM1_ESM.docx]

**Additional file 1: Model Input Data**

Table S1 – Assumed herd protection effects for vaccinated and unvaccinated age cohorts

|  | **Vaccinated** | | | **Unvaccinated** | | |
| --- | --- | --- | --- | --- | --- | --- |
| Age group | study estimates | Source | Model input | study estimates | Source | Model input |
| <2-3 months | 61% | [1, 2] | 30% | NA |  | 0 |
| 3 mo -1 year | 37-65%* | [3] | 25% | NA | [1-5] | 0 |
| 1-2 years | 37-65%* |  | 25% | 33-72% |  | 28% |
| 2-3 years | 37-65%* |  | 25% | 38-62% |  | 28% |
| 3-4 years | 37-65%* |  | 25% | 36-65% |  | 28% |
| 4-5 years | 37-65%* |  | 25% | 25-65% |  | 28% |
| 6-10 years | NA |  | 0 | 39-61% |  | 25% |
| 10-14 years | NA |  | 0 | 50-55% |  | 25% |

* age-group not specified, average of ages < 5

**Intussusception length of hospital stay**

Administrative hospital discharge data were retrospectively collected from the Dutch National Medical Registration (LMR) database. The LMR database covers around 90% of the total Dutch population of 17 million people. It contains anonymized data on hospital admissions, outpatient consultation and emergency department visits including discharge diagnoses according to the International Classification of Diseases (ICD) codes, date of admission and discharge, patient age and gender. For diagnostic coding, the Ninth Revision (ICD-9) was used up to 2012 and ICD-10 thereafter. A validation study showed high accuracy of coding and concluded that the discharge data are generally of high quality [6].

For the period from 1 January 2008 to 31 December 2012, we extracted all cases with a primary or secondary discharge diagnosis of intussusception (ICD-9 CM code 560.0) in children aged between 0 and 12 months (i.e. children aged 0–35 months). A total of 276 cases were retrieved. The average length of stay was 2.11 days and 95% of patients were discharged within 6 days of hospitalization. For the complicated IS cases, we assumed their hospital stay represented the upper 5% of the length of stay distribution which was > 6 days, 3 times more than the average length of stay. We thus assumed a three times longer hospital stay for complicated IS cases, compared to uncomplicated IS cases. A similar assumption was applied for utility losses, such that QALY losses for complicated IS were three times that of uncomplicated IS.

**RotaFam study**

This is a prospective community-based household study on AGE occurrence and transmission [7]. Households with at least one child aged less than two years were randomly selected from municipal registries and invited to participate in the study. Participating households kept a digital symptom diary by means of an interactive smartphone App for each household member during ten consecutive weeks between January and May 2016 or 2017. Occurrence of AGE, which was detected based on symptom entries through built-in algorithms in the App, triggered additional disease questionnaires and a stool sample request for virological examination. The disease questionnaires included a severity score, items on healthcare usage and parental work-loss. All samples were tested by multiplex PCR for presence of norovirus, rotavirus, astrovirus and adenovirus 40/41. Data from the 2016 season on rotavirus AGE episodes in children were used to estimate parental productivity losses due to work-absence for community (i.e. mild) and GP attended (i.e. moderate) episodes.

We identified 28 community episodes of rotavirus AGE in children < 15 years. Of these, eight were GP attended. In three episodes (11%), a caregiver took time off from work varying between 0.5 and 2 days. These sick children were aged < 2 years and two had also attended the GP.

Thus, in one out of 20 episodes without medical care a caregiver took time off from work (ie. 1 day in 5% of cases). For two out of eight GP-attended episodes, a caregiver took time off from work (ie. 0.5-2 days in 25% of cases).
