# Supplementary material for: Updated cost-effectiveness and risk-benefit analysis of two infant rotavirus vaccination strategies in a high-income, low-endemic setting
Source: BMC Med. 2018 Sep 10;16:168. doi: 10.1186/s12916-018-1134-3 (PMC6130096; doi:10.1186/s12916-018-1134-3)
Supplement: Supplementary file 2 — Additional results. (DOCX 71 kb) [file 12916_2018_1134_MOESM2_ESM.docx]

**Additional file 2: Additional results**

Table S2 – Targeted vaccination compared to no vaccination: Baseline assumptions and applied sensitivity and scenario analyses

| **Scenario** | **∆ QALY** | **∆ societal cost**  **(in mio. €)^a^** | **ICER (€/QALY) - Societal perspective** | **∆ healthcare cost**  **(in mio. €)^a^** | **ICER**  **(€/QALY) - Healthcare payer perspective** | **Induced IS / complicated IS cases** | **Induced IS: prevented fatal cases** | **Induced IS: prevented hospitalized cases** | |
| --- | --- | --- | --- | --- | --- | --- | --- | --- | --- |
|  | Mean  (95% CI) | Mean  (95% CI) | Mean  (95% CI) | Mean  (95% CI) | Mean  (95% CI) | Mean | Mean  (95% CI) | Mean  (95% CI) | |
| Baseline | 1139  (426-2022) | -17.0  (-20.8--13.6) | cost-saving (cs-cs) | -11.1  (-14.2--8.4) | cost-saving  (cs-cs) | 4.61/0.22 | 1:21  (1:12-1:33) | 1:1707  (1:1494-1:1920) | |
| *Sensitivity and scenario analyses:* | | | | | | | | |  |
| *w.r.t. assumed intussusception (IS) risk (in baseline 1:50,000) and complicated IS (in baseline 4.8%)* | | | | | | | | |  |
| IS risk: 1:20,000 | 1139  (426-2022) | -17.0  (-20.8--13.6) | cost-saving (cs-cs) | -11.1  (-14.2- -8.4) | cost-saving  (cs-cs) | 11.53/0.55 | 1:9  (1:5-1:13) | 1:683  (1:598-1:768) | |
| IS risk: 1:100,000 | 1139  (426-2022) | -17.0  (-20.8--13.6) | cost-saving (cs-cs) | -11.1  (-14.2--8.4) | cost-saving  (cs-cs) | 2.31/0.11 | 1:43  (1:23-1:66) | 1:3414  (1:2988-1:3840) | |
| Complicated IS 0% | 1139  (426-2022) | -17.0  (-20.8--13.6) | cost-saving (cs-cs) | -11.1  (-14.2--8.4) | cost-saving  (cs-cs) | 4.61/0.00 | 1:21  (1:12-1:33) | 1:1707  (1:1494-1:1920) | |
| Complicated IS 9.6% | 1139  (426-2022) | -17.0  (-20.8--13.6) | cost-saving (cs-cs) | -11.1  (-14.2--8.4) | cost-saving  (cs-cs) | 4.61/0.44 | 1:21  (1:12-1:33) | 1:1707  (1:1494-1:1920) | |
| *w.r.t. assumed hospitalization rate* | | | | | | | | |  |
| Lower hospitalization rate (*75%) | 870  (283-1561) | -11.5  (-14.5--8.9) | cost-saving (cs-cs) | -6.9  (-9.2--4.8) | cost-saving  (cs-cs) | 4.61/0.22 | 1:16  (1:9-1:25) | 1:1,260  (1:1121-1:1440) | |
| Higher hospitalization rate (*125%) | 1414  (566-2482) | -22.4  (-18.3--27.1) | cost-saving (cs-cs) | -15.3  (-11.9--19.2) | cost-saving  (cs-cs) | 4.61/0.22 | 1:27  (1:15-1:41) | 1:2134  (1:1868-1:2400) | |
| *w.r.t. hospitalization costs* | | | | | | | | |  |
| Lower hospitalization costs (*75%) | 1139  (426-2022) | -13.0  (-16.0--10,2) | cost-saving (cs-cs) | -7.1 (-9.4—5.0) | cost-saving (cs-cs) | 4.61/0.22 | 1:21  (1:12-1:33) | 1:1707  (1:1494-1:1920) | |
| Higher hospitalization costs (*125%) | 1139  (426-2022) | -21.0 (-25.5—17.0) | cost-saving (cs-cs) | -15.1 (-19.0—11.7) | cost-saving (cs-cs) | 4.61/0.22 | 1:21  (1:12-1:33) | 1:1707  (1:1494-1:1920) | |
| *w.r.t. productivity losses* | | | | | | | | |  |
| caregiver work absence as in [8] **^b^** | 1139  (426-2022) | -16.8  (-20.5--13.6) | cost-saving (cs-cs) | -11.1  (-14.2--8.4) | cost-saving  (cs-cs) | 4.61/0.22 | 1:21  (1:12-1:33) | 1:1707  (1:1494-1:1920) | |
| *w.r.t. assumed QALY losses (in baseline: 0.0011, 0.0022, 0.0034 and 0.0026 for mild, moderate, severe and nosocomical infections, respectively)* | | | | | | | | |  |
| QALY losses as in [8] **^c^** | 1134  (421-2017) | -17.0  (-20.8--13.6) | cost-saving (cs-cs) | -11.1  (-14.2--8.4) | cost-saving  (cs-cs) | 4.61/0.22 | 1:21  (1:12-1:33) | 1:1707  (1:1494-1:1920) | |
| higher QALY losses [9] **^d^** | 1146  (433-2029) | -17.0  (-20.8--13.6) | cost-saving (cs-cs) | -11.1  (-14.2--8.4) | cost-saving  (cs-cs) | 4.61/0.22 | 1:21  (1:12-1:33) | 1:1707  (1:1494-1:1920) | |
| *w.r.t. assumed discount rate (3% for costs and effects in baseline)* | | | | | | | | |  |
| Discount rate: 0% effects & costs | 2385  (792-4363) | -22.6  (-27.6--18.2) | cost-saving (cs-cs) | -14.8  (-18.9--11.2) | cost-saving  (cs-cs) | 4.61/0.22 | 1:21  (1:12-1:33) | 1:1707  (1:1494-1:1920) | |
| Discount rate: 2% effects & costs | 1435  (528-2560) | -18.6  (-22.7--14.9) | cost-saving (cs-cs) | -12.2  (-15.6--9.2) | cost-saving  (cs-cs) | 4.61/0.22 | 1:21  (1:12-1:33) | 1:1707  (1:1494-1:1920) | |
| Discount rate: 4% effects & costs | 916  (349-1615) | -15.5  (-19.0--12.5) | cost-saving (cs-cs) | -10.1  (-13.0--7.6) | cost-saving  (cs-cs) | 4.61/0.22 | 1:21  (1:12-1:33) | 1:1707  (1:1494-1:1920) | |
| Dutch Discount rates (effects (1.5%) & costs (4%)) [10] | 1620  (583-2909) | -15.5  (-19.0--12.5) | cost-saving (cs-cs) | -10.1  (-13.0--7.6) | cost-saving  (cs-cs) | 4.61/0.22 | 1:21  (1:12-1:33) | 1:1707  (1:1494-1:1920) | |
| *w.r.t. older age at first infection and consequently slightly lower probability of seeking medical care^e^* | | | | | | | | |  |
| 50% of 0-1 year olds were assumed to be 1-2 years old when infected | 1052  (383-1873) | -14.3  (-17.8—11.3) | cost-saving (cs-cs) | -9.0  (-11.9—6.5) | cost-saving  (cs-cs) | 4.61/0.22 | 1:20  (1:11-1:31) | 1:1532  (1:1340-1:1725) | |
| 75% of 0-1 year olds were assumed to be 1-2 years old when infected | 9968  (335-1773) | -12.7  (-15.9—9.8) | cost-saving (cs-cs) | -7.7  (-10.4--5.4) | cost-saving  (cs-cs) | 4.61/0.22 | 1:19  (1:10-1:29) | 1:1420  (1:1239-1:1602) | |
| *w.r.t. assumed vaccine coverage (in baseline: 86% coverage)* | | | | | | | | |  |
| Vaccine coverage: 75% | 1113  (406-1987) | -14.8  (-18.1--11.8) | cost-saving (cs-cs) | -9.6  (-12.4--7.3) | cost-saving  (cs-cs) | 4.02/0.19 | 1:24  (1:13-1:37) | 1:1707  (1:1494-1:1920)) | |

1. Note: negative costs are savings
2. In the earlier model the assumed hours of work loss for mild cases were: 0.93; 1.36; 0.84 days for ages 0 to 4; 5 to 9 and 10 to 14 years respectively, versus in the baseline: 1 day (~8 hours) in 5% of episodes for children under the age of 10 and for children > 10 years of age work loss estimates were reduced by 50%. In the earlier model the assumed hours of work loss for moderate cases were: 1.35; 1.98; 1.23 for ages 0 to 4; 5 to 9 and 10 to 14 years respectively, versus in the baseline: 0.5 - 2 days in 25% of episodes for children under the age of 10 and for children > 10 years of age work loss estimates were reduced by 50%.
3. Bruijning et al.[8] used 0.0011, 0.0022, 0.0022 and 0.0020 for mild, moderate, severe and nosocomial infections, respectively.
4. We included slightly higher QALY losses based on the sensitivity analysis of Marlow et al. (for hospitalizations 0.0039 vs 0.0030 in the baseline and for GP visits 0.0030 vs 0.0022 in the baseline)[9])
5. On average 21.5% of the 0-1 years old would require a GP visit, whereas only 18.5% of 1-4 years old (Table 1). As the GP has a gatekeeper function in the Netherlands, we modelled that on average 27.6% of the 0-1 year olds visiting a GP would require hospitalization, whereas this would be only 16.4% if 1-4 years old. These averages were derived from the baseline simulations results.

CI: confidence interval; cs: cost-saving; (cs-cs): 95%CI limits both cost-saving; IS: intussusception; S.A.: Sensitivity analysis or scenario analysis.

Table S3 – Universal vaccination compared to no vaccination: Baseline assumptions and applied sensitivity and scenario analyses

| **Scenario** | **∆ QALY** | | | | **∆ societal cost**  **(in mio. €)^a^** | **ICER (€/QALY) - Societal perspective** | **∆ healthcare cost**  **(in mio. €)^a^** | **ICER**  **(€/QALY) - Healthcare payer perspective** | **Induced IS / complicated IS cases** | | **Induced IS: prevented fatal cases** | **Induced IS: prevented hospitalized cases** |  |
| --- | --- | --- | --- | --- | --- | --- | --- | --- | --- | --- | --- | --- | --- |
|  | Mean  (95% CI) | | | | Mean  (95% CI) | Mean  (95% CI) | Mean  (95% CI) | Mean  (95% CI) | Mean | Mean  (95% CI) | | Mean  (95% CI) |  |
| Baseline | 1907  (1114-2915) | | | | 98  (74-116) | 51,277  (29,259-94,686) | 137  (126-148) | 72,021  (45,102-126,919) | 58.4 / 2.8 | 1:1.8  (1:1.0-1:2.8) | | 1:685  (1:603-1:767) |  |
| *Sensitivity and scenario analyses:* | | | | | | | | | | | | |  |
| *w.r.t. assumed intussusception (IS) risk (in baseline 1:50,000) and complicated IS (in baseline 4.8%)* | | | | | | | | | | | | |  |
| IS risk: 1:20,000 | | 1907  (1114-2915) | | | 98  (74-116) | 51,350  (29,302-94,814) | 137  (126-148) | 72,090  (45,144-127,054) | 146.0/7.0 | 1:0.7  (1:0.4-1:1.1) | | 1:274  (1:241-1:307) |  |
| IS risk: 1:100,000 | | 1908  (1114-2916) | | | 98  (74-116) | 51,253  (29,245-94,643) | 137  (126-148) | 71,998  (45,088-126,874) | 29.0/1.4 | 1:3.5  (1:1.9-1:5.6) | | 1:1370  1:1206-1:1533 |  |
| Complicated IS 0% | | 1908  (1114-2915) | | | 98  (74-116) | 51,270  (29,255-94,674) | 137  (126-148) | 72,014  (45,098-126,906) | 58.4 / 0.0 | 1:1.8  (1:1.0-1:2.8) | | 1:685  (1:603-1:767) |  |
| Complicated IS 9.6% | | 1907  (1114-2915) | | | 98  (74-116) | 51,284  (29,263-94,697) | 137  (126-148) | 72,027  (45,106-126,931) | 58.4 / 5.6 | 1:1.8  (1:1.0-1:2.8) | | 1:685  (1:603-1:767) |  |
|  | |  | | |  |  |  |  |  |  | |  |  |
| *w.r.t. assumed hospitalization rate* | | | | | | | | | | | | |  |
| Lower hospitalization rate (*75%) | | 1608  (949-2419) | | | 124  (101-140)) | 76,831  (47,406-135,892) | 157  (148-166) | 97,654  (63,262-169,264)) | 58.4 / 2.8 | 1:1.3  (1:0.7-1:2.1) | | 1:514  (1:452-1:575) |  |
| Higher hospitalization rate (*125%) | | 2213  (1262-3435)) | | | 72  (47-93) | 32,581  (16,434-63,943) | 118  (104-131) | 53,214  (32,257-96,889) | 58.4 / 2.8 | 1:2.2  (1:1.2-1:3.5) | | 1:856  (1:754-1:958) |  |
| *w.r.t. hospitalization costs* | | | | | | | | | | | | |  |
| Lower hospitalization costs (*75%) | | 1907  (1114-2915) | | | 116  (94-133) | 61,023  (36,124-110,191) | 156  (147-165) | 81,767  (51,898-142,952) | 58.4 / 2.8 | 1:1.8  (1:1.0-1:2.8) | | 1:685  (1:603-1:767) |  |
| Higher hospitalization costs (*125%) | | 1907  (1114-2915) | | | 79  (54-100) | 41,531  (22,321-79,488) | 119  (105-132) | 62,275  (38,511-111,246) | 58.4 / 2.8 | 1:1.8  (1:1.0-1:2.8) | | 1:685  (1:603-1:767) |  |
| *w.r.t. productivity losses* | | | | | | | | | | | | |  |
| Higher productivity losses (* 200%) | | 1907  (1114-2915) | | | 60  (20-88) | 31,659  (9,010-65,904) | 137  (126-148) | 72,027  (45,106-126,931) | 58.4 / 2.8 | 1:1.8  (1:1.0-1:2.8) | | 1:685  (1:603-1:767) |  |
| Caregiver work loss ass in [8] **^b^** | | 1907  (1114-2915) | | | 99  (83-114) | 51,746  (30,863-94,173) | 137  (126-148) | 72,021  (45,102-126,919) | 58.4 / 2.8 | 1:1.8  (1:1.0-1:2.8) | | 1:685  (1:603-1:767) |  |
| *w.r.t. assumed QALY losses (in baseline: 0.0011, 0.0022, 0.0034 and 0.0026 for mild, moderate, severe and nosocomical infections, respectively)* | | | | | | | | | | | | |  |
| QALY losses as in [8] **^c^** | | 1875  (1083-2882) | | | 98  (74-116) | 52,165  (29,599-97,158) | 137  (126-148) | 73,268  (45,633-130,334) | 58.4 / 2.8 | 1:1.8  (1:1.0-1:2.8) | | 1:685  (1:603-1:767) |  |
| Higher QALY losses [9] **^d^** | | 2144  (1332-3179) | | | 98  (74-116) | 45,620  (26,919-80,560) | 137  (126-148) | 64,075  (41,500-106,594) | 58.4 / 2.8 | 1:1.8  (1:1.0-1:2.8) | | 1:685  (1:603-1:767) |  |
| *w.r.t. assumed discount rate (3% for costs and effects in baseline)* | | | | | | | | | | | | |  |
| Discount rate: 0% effects & costs | | 3525  (1715-5891) | | | 126  (95-150) | 35,747  (18,828-77,207) | 178  (163-192) | 50,537  (29,130-104,921) | 58.4 / 2.8 | 1:1.8  (1:1.0-1:2.8) | | 1:685  (1:603-1:767) |  |
| Discount rate: 2% effects & costs | | 2294  (1279-3612) | | | 106  (80-126) | 46,192  (25,696-88,889) | 149  (137-161) | 65,025  (39,734-119,999) | 58.4 / 2.8 | 1:1.8  (1:1.0-1:2.8) | | 1:685  (1:603-1:767) |  |
| Discount rate: 4% effects & costs | | 1612  (976-2416) | | | 91  (69-108) | 56,223  (32,908-101,064) | 127  (117-137) | 78,785  (50,448-133,670) | 58.4 / 2.8 | 1:1.8  (1:1.0-1:2.8) | | 1:685  (1:603-1:767) |  |
| Dutch Discount rates (effects (1.5%) & costs (4%)) [10] | | 2534  (1382-4041) | | | 91  (69-108) | 35,773  (19,780-70,545) | 127  (117-137) | 50,128  (30,299-94,832) | 58.4 / 2.8 | 1:1.8  (1:1.0-1:2.8) | | 1:685  (1:603-1:767) |  |
| *w.r.t. older age at first infection and consequently slightly lower probability of seeking medical care^e^* | | | | | | | | | | | | |  |
| 50% of 0-1 year olds were assumed to be 1-2 years old when infected | | | 1785  (1029-2742) | 113  (90-130) | | 63,147  (37,271-115,558) | 148  (138-158) | 83,144  (52,799-146,342) | 58.4/2.8 | 1:1.6  (1:0.9-1:2.6) | | 1:598  (1:528-1:669) | |
| 75% of 0-1 year olds were assumed to be 1-2 years old when infected | | | 1706  (984-2608) | 124  (102-141) | | 72,777  (44,198-131,290) | 157  (148-166) | 92,015  (58,897-161,918) | 58.4/2.8 | 1:1.5  (1:0.8-1:2.4) | | 1:528  (1:465-1:592) | |
| *w.r.t. assumed herd immunity (in baseline herd immunity was considered)* | | | | | | | | | | | | |  |
| No herd immunity | | 1690  (916-2680) | | | 115  (96-131) | 68,092  (39,790-131,501) | 148  (138-158) | 87,833  (53,677-166,304) | 58.4 / 2.8 | 1:1.7  (1:0.9-1:2.8) | | 1:603  (1:527-1:678) |  |
| Lower herd immunity (*50%) | | 1798  (1016-2800) | | | 106  (85-124) | 59,188  (34,260-111,126) | 143  (132-153) | 79,465  (49,224-144,298) | 58.4 / 2.8 | 1:1.8  (1:0.9-1:2.8) | | 1:644  (1:566-1:722) |  |
| Higher herd immunity (*150%) | | 2017  (1212-3045) | | | 89  (64-109) | 44,204  (24,791-81,325) | 132  (120-143) | 65,356  (41,380-112,987) | 58.4 / 2.8 | 1:1.8  (1:1.0-1:2.9) | | 1:726  (1:640-1:812) |  |
| *for an alternative universal vaccination (in baseline: vaccine coverage of 86% & herd immunity & vaccine costs of €75/child)* | | | | | | | | | | | | |  |
| Vaccine coverage 60% & **no** herd immunity & market price (€135.3/child) | | 1480  (730-2443) | | | 176  (163-188) | 119,191  (70,488-244,692) | 200  (193-207) | 134,920  (80,702-275,744) | 40.8/2.0 | 1:2.4  (1:1.3-1:3.8) | | 1:603  (1:527-1:678) |  |
| *w.r.t. vaccine cost per child, excluding application costs (in baseline vaccine costs of €75/child)* | | | | | | | | | | | | |  |
| Vaccine costs of €67.5 | | 1907  (1114-2915) | | | 81  (57-99) | 42,304  (23,270-79,865) | 120  (109-131) | 63,048  (39,266-111,667) | 58.4 / 2.8 | 1:1.8  (1:1.0-1:2.8) | | 1:685  (1:603-1:767) |  |
| Vaccine costs of €60.0 | | 1907  (1114-2915) | | | 64  (40-82) | 33,331  (16,809-64,499) | 103  (92-114) | 54,075  (33,482-96,411) | 58.4 / 2.8 | 1:1.8  (1:1.0-1:2.8) | | 1:685  (1:603-1:767) |  |
| Vaccine costs of €52.5 | | 1907  (1114-2915) | | | 46  (23-65) | 24,358  (9,958-49,675) | 86  (75-97) | 45,102  (27,586-81,051) | 58.4 / 2.8 | 1:1.8  (1:1.0-1:2.8) | | 1:685  (1:603-1:767) |  |
| Vaccine costs of €45.0 | | 1907  (1114-2915) | | | 29  (6-48) | 15,385  (2,750-34,992) | 69  (58-80) | 36,129  (21,511-66,081) | 58.4 / 2.8 | 1:1.8  (1:1.0-1:2.8) | | 1:685  (1:603-1:767) |  |
| Vaccine costs of €37.5 | | 1907  (1114-2915) | | | 12  (-11-31) | 6,412  (ca -21,296) | 52  (41-63) | 27,156  (15,467-50,957) | 58.4 / 2.8 | 1:1.8  (1:1.0-1:2.8) | | 1:685  (1:603-1:767) |  |
| Vaccine costs of €30.0 | | 1907  (1114-2915) | | | -5  (-28 - 14) | cost-saving  (cs - 2,561) | 35  (23-45) | 18,183  (9,298-36,179) | 58.4 / 2.8 | 1:1.8  (1:1.0-1:2.8) | | 1:685  (1:603-1:767) |  |
| Vaccine costs of €22.5 | | 1907  (1114-2915) | | | -22  (-46 - -3) | cost-saving  (cs- cs) | 18  (6-28) | 9,209  (2,758-21,476) | 58.4 / 2.8 | 1:1.8  (1:1.0-1:2.8) | | 1:685  (1:603-1:767) |  |
| Vaccine costs of €15.0 | | 1907  (1114-2915) | | | -39  (-63 - -21) | cost-saving  (cs - cs) | 0.5  (-11 - 11) | 236  (cs – 7,623) | 58.4 / 2.8 | 1:1.8  (1:1.0-1:2.8) | | 1:685  (1:603-1:767) |  |

1. Note: negative costs are savings
2. In the earlier model the assumed hours of work loss for mild cases were: 0.93; 1.36; 0.84 days for ages 0 to 4; 5 to 9 and 10 to 14 years respectively versus in the baseline: 1 day (~8 hours) in 5% of episodes for children under the age of 10 and for children > 10 years of age work loss estimates were reduced by 50%. In the earlier model the assumed hours of work loss for moderate cases were: 1.35; 1.98; 1.23 for ages 0 to 4; 5 to 9 and 10 to 14 years respectively versus in the baseline: 0.5 – 2 days in 25% of episodes for children under the age of 10 and for children > 10 years of age work loss estimates were reduced by 50%.
3. Bruijning et al.[8] used 0.0011, 0.0022, 0.0022 and 0.0020 for mild, moderate, severe and nosocomial infections, respectively.
4. We included slightly higher QALY losses based on the sensitivity analysis of Marlow et al. (for hospitalizations 0.0039 vs 0.0030 in the baseline and for GP visits 0.0030 vs 0.0022 in the baseline)[9]).
5. On average 21.5% of the 0-1 years old would require a GP visit, whereas only 18.5% of 1-4 years old (Table 1). As the GP has a gatekeeper function in the Netherlands, we modelled that on average 27.6% of the 0-1 year olds visiting a GP would require hospitalization, whereas this would be only 16.4% if 1-4 years old. These averages were derived from the baseline simulations results.

Used abbreviations: CI: confidence interval; cs: cost-saving; (cs-cs): 95%CI limits both cost-saving; IS: intussusception; S.A.: Sensitivity analysis or scenario analysis.

Table S4 – Universal vaccination compared to target vaccination: Baseline assumptions and applied sensitivity and scenario analyses

| **Scenario** | **∆ QALY** | | **∆ societal cost**  **(in mio. €)^a^** | **ICER (€/QALY) - Societal perspective** | **∆ healthcare cost**  **(in mio. €)** | | **ICER**  **(€/QALY) - Healthcare payer perspective** | **Induced IS / complicated IS cases** | | | **Induced IS: prevented fatal cases** | **Induced IS: prevented hospitalized cases** |
| --- | --- | --- | --- | --- | --- | --- | --- | --- | --- | --- | --- | --- |
|  | Mean  (95% CI) | | Mean  (95% CI) | Mean  (95% CI) | Mean  (95% CI) | Mean  (95% CI) | | Mean | | Mean  (95% CI) | | Mean  (95% CI) |
| Baseline | 769  (561-1003) | | 115  (94-131) | 149,282  (101,101-220,113) | 148  (139-157) | 193,084  (142,802-271,403) | | 53.8/ 2.6 | 1:0.09  (1:0.02-1:0.2) | | | 1:597  (1:526-1:668) |
| *Sensitivity and scenario analyses:* | | | | | | | | | | | | |
| *w.r.t. vaccine cost per child, excluding application costs (in baseline vaccine costs of €75 were assumed)* | | | | | | | | | | | | |
| Vaccine costs of €67.5 | | 769  (561-1003) | 98  (77-114) | 127,021  (82,989-190,334) | 131  (122-140) | 170,823  (125,657-241,489) | | 53.8/ 2.6 | 1:0.09  (1:0.02-1:0.2) | | | 1:597  (1:526-1:668) |
| Vaccine costs of €60.0 | | 769  (561-1003) | 81  (59-97) | 104,760  (65,365-161,065) | 114  (105-123) | 148,562  (108,357-211,313) | | 53.8/ 2.6 | 1:0.09  (1:0.02-1:0.2) | | | 1:597  (1:526-1:668) |
| Vaccine costs of €52.5 | | 769  (561-1003) | 63  (42-79) | 82,500  (47,090-131,537) | 97  (88-106) | 126,301  (91,196-181,116) | | 53.8/ 2.6 | 1:0.09  (1:0.02-1:0.2) | | | 1:597  (1:526-1:668) |
| Vaccine costs of €45.0 | | 769  (561-1003) | 46  (25-62) | 60,239  (28,385-101,948) | 80  (71-89) | 104,040  (73,947-151,104) | | 53.8/ 2.6 | 1:0.09  (1:0.02-1:0.2) | | | 1:597  (1:526-1:668) |
| Vaccine costs of €37.5 | | 769  (561-1003) | 29  (8-45) | 37,978  (9,277-73,241) | 63  (54-72) | 81,779  (56,779-120,988) | | 53.8/ 2.6 | 1:0.09  (1:0.02-1:0.2) | | | 1:597  (1:526-1:668) |
| Vaccine costs of €30.0 | | 769  (561-1003) | 19  (-9-28) | 15,717  (cost-saving-44,867) | 46  (37-55) | 59,519  (39,427-90,783) | | 53.8/ 2.6 | 1:0.09  (1:0.02-1:0.2) | | | 1:597  (1:526-1:668) |
| Vaccine costs of €22.5 | | 769  (561-1003) | -5  (-26-11) | cost-saving  (cost-saving-16,466) | 29  (20-37) | 37,528  (21,440-61,339) | | 53.8/ 2.6 | 1:0.09  (1:0.02-1:0.2) | | | 1:597  (1:526-1:668) |
| Vaccine costs of €15.0 | | 769  (561-1003) | -22  (-43 - -6) | cost-saving  (cost-saving - cost-saving) | 12  (2-20) | 14,997  (2,738-32,159) | | 53.8/ 2.6 | 1:0.09  (1:0.02-1:0.2) | | | 1:597  (1:526-1:668) |

1. Note: negative costs are savings

Note2: No sensitivity analyses on vaccine costs were applied for the target vaccination strategy, as this scenario was already cost-saving at current market prices of €135.32, for both perspectives. By lowering the vaccine costs, the savings would become larger.

Figure S1: Mean ICER for targeted* and universal vaccination using a societal perspective (black square/black line) and healthcare payer perspective (red dots/red line), for different vaccine costs.

*No sensitivity analyses on vaccine costs were applied for the targeted vaccination strategy, as this scenario was already cost-saving at current market prices of €135.32, for both perspectives. By lowering the vaccine costs, the savings would become larger.

Note: In case of universation vaccination strategy is vaccination cost-saving at vaccine costs of €32/child using a societal perspective (SP) and at €14.8/child using a healthcare payer perspective (HP). For full details see Table A.3.

**References**

1. Prelog M, Gorth P, Zwazl I, Kleines M, Streng A, Zlamy M, Heinz-Erian P, Wiedermann U: **Universal Mass Vaccination Against Rotavirus: Indirect Effects on Rotavirus Infections in Neonates and Unvaccinated Young Infants Not Eligible for Vaccination**. *J Infect Dis* 2016, **214**(4):546-555.

2. Atchison CJ, Stowe J, Andrews N, Collins S, Allen DJ, Nawaz S, Brown D, Ramsay ME, Ladhani SN: **Rapid Declines in Age Group-Specific Rotavirus Infection and Acute Gastroenteritis Among Vaccinated and Unvaccinated Individuals Within 1 Year of Rotavirus Vaccine Introduction in England and Wales**. *J Infect Dis* 2016, **213**(2):243-249.

3. Krishnarajah G, Demissie K, Lefebvre P, Gaur S, Sheng Duh M: **Clinical and cost burden of rotavirus infection before and after introduction of rotavirus vaccines among commercially and Medicaid insured children in the United States**. *Hum Vaccin Immunother* 2014, **10**(8):2255-2266.

4. Kaufman HW, Chen Z: **Trends in Laboratory Rotavirus Detection: 2003 to 2014**. *Pediatrics* 2016, **138**(4).

5. Sabbe M, Berger N, Blommaert A, Ogunjimi B, Grammens T, Callens M, Van Herck K, Beutels P, Van Damme P, Bilcke J: **Sustained low rotavirus activity and hospitalisation rates in the post-vaccination era in Belgium, 2007 to 2014**. *Euro Surveill* 2016, **21**(27).

6. Paas G, Veenhuizen K: **Onderzoek naar de betrouwbaarheid van de Landelijke Medische Registratie (LMR). [Research on the reliability of the National Medical Registration (LMR)]**. In*.* Utrecht: Prismant; 2002: 1-22.

7. Quee F, de Hoog MLA, Schuurman R, Bruijning-Verhagen P: **A comparison of incidence, transmission and burden of norovirus and rotavirus infections among young families in the Netherlands**. In: *5th European Expert Meeting on Rotavirus Vaccination: March 20-22nd, 2017 2017; Utrecht*; 2017: 83-84.

8. Bruijning-Verhagen P, Mangen MJ, Felderhof M, Hartwig NG, van Houten M, Winkel L, de Waal WJ, Bonten MJ: **Targeted rotavirus vaccination of high-risk infants; a low cost and highly cost-effective alternative to universal vaccination**. *BMC Med* 2013, **11**:112.

9. Marlow R, Finn A, Trotter C: **Quality of life impacts from rotavirus gastroenteritis on children and their families in the UK**. *Vaccine* 2015, **33**(39):5212-5216.

10. ZIN: **Kostenhandleiding: Methodologie van kostenonderzoek en referentieprijzen voor economische evaluaties in de gezondheidszorg**. In*.*, vol. Geactualiseerde versie 2015: Zorginstituut Nederland (ZIN); 2015.
